# Plumage Response of Young Turkeys to Diets with Increased Methionine to Lysine Ratios at Three Dietary Arginine Levels

**DOI:** 10.3390/ani12020172

**Published:** 2022-01-12

**Authors:** Emilia Mróz, Jan Jankowski, Marek Skowroński, Dariusz Mikulski

**Affiliations:** Department of Poultry Science and Apiculture, University of Warmia and Mazury in Olsztyn, Oczapowskiego 5, 10-718 Olsztyn, Poland; emilia.mroz@uwm.edu.pl (E.M.); janj@uwm.edu.pl (J.J.); skowronskimarek97@gmail.com (M.S.)

**Keywords:** feather growth, turkey, methionine, arginine

## Abstract

**Simple Summary:**

An adequate supply of essential amino acids through the diet is critical for maintaining a fast growth rate, good health, and proper immune function as well as feather-cover development in poultry species. Feathers contain about 90% of protein, therefore the optimal ratios of limiting amino acids, in particular the sulfur-containing amino acids are indicated as necessary for the synthesis of feather keratin. This study evaluated the effects of different dietary methionine (Met) and arginine (Arg) levels on plumage development in young turkeys. An increased supply of sulfur-containing amino acids via supplemental Met promoted feather growth in turkeys at 16 weeks of age. Different concentrations of Arg (90%, 100%, and 110% of lysine content) had no influence on plumage development. The data on feather growth can contribute to a better understanding of the amino acid requirements in modern commercial turkey-farming systems.

**Abstract:**

A 2 × 3 factorial experiment was conducted to evaluate the effects of two dietary methionine levels (Met; 30% and 45% of Lys content) and three arginine levels (Arg; 90%, 100%, and 110% of Lys content) on plumage development in 4- and 16-week-old female turkeys. One-day-old turkey poults were assigned to six groups (eight replicate pens per group and 18 birds per pen) and fed experimental diets containing 1.6%, 1.5%, 1.3%, and 1.0% of Lys in four successive four-week periods. After weeks 4 and 16 of feeding, eight turkeys per group were selected for plumage evaluation. Feathers were collected from the outer side of one thigh and from an area of 4 cm^2^ in the interscapular region. Plumage was evaluated based on an established pattern of five feather development stages in turkeys, from stage I (pinfeathers covered in sheaths) to stage V (mature feathers). An increase in the Met inclusion rate to 45% of Lys content had no significant effect on feather growth in 4-week-old turkeys, but it accelerated the development of feathers in 16-week-old birds. A lower percentage of stage II (*p* = 0.035), stage III (*p* = 0.019), and stage IV (*p* = 0.003) immature feathers, and a higher percentage of stage V (mature) feathers (*p* = 0.001) were observed. Methionine exerted a greater effect on the development of thigh feathers (*p* = 0.001) than interscapular feathers (*p* = 0.074). Unlike Met, different Arg concentrations had no influence on plumage development in turkeys. Overall, the present results indicate that supplemental Met has a potential for accelerating feather development in 16-week-old turkeys via an increased supply of total sulfur amino acids.

## 1. Introduction

Feather growth, structure, and patterns of molting are important characteristics of poultry in commercial production [1]. Healthy plumage provides thermal insulation and protects skin against damage and infections, thus improving the microbiological status of carcasses. Poorly feathered birds have an increased requirement for maintenance energy [2], which is associated with higher feeding costs [3] and the quality parameters of their carcasses are poorer [4]. It has been suggested that feather traits, including fault bars, and feather growth can be used as indicators of the nutritional or physiological status in chickens [5]. Attempts have recently been made to assess the welfare of domestic poultry based on measurements of the stress hormone corticosterone and metabolites present in growing feathers [6,7,8,9].

Feathers contain approximately 90% of protein, therefore the optimal ratios of limiting amino acids, in particular the sulfur-containing amino acids methionine (Met) and cystine (Cys), are indicated as necessary for the synthesis of feather keratin [4,10,11,12,13]. The sulfur-containing amino acid Cys is the major amino acid involved in the synthesis of feather keratin, which suggests a high dietary requirement of this amino acid [1,14,15]. Another sulfur-containing amino acid, Met, is involved through conversion to Cys, which occurs in both the liver and the feather follicle [16,17,18]. Methionine is considered to be the main nutritional factor affecting feather cover and feather length [19]. A beneficial influence of total sulfur amino acids (TSAA) has been observed in several experiments on chickens, but feather cover or feather growth have not been examined [20,21,22].

Good feather growth and maintenance, and the contribution of adequate nutrition to feather synthesis are often disregarded in poultry research and industry. Several studies revealed a positive effect of dietary Met on feather cover and feather weight in ducks [19,23]. In an experiment performed by Kubińska et al. [24], increased dietary Met concentration from 25 to 40% of Lys content did not accelerate plumage development or maturation in 4- and 8-week-old female turkeys. Our recent research involving turkeys has focused on the role of amino acids such as Lys, Arg, and Met in the regulation of different metabolic processes including protein synthesis as well as immune responses, hormone secretion, and antioxidant activity, but no attention was then paid to feather cover [25,26,27,28,29,30,31]. Jankowski et al. [27] reported that body weight gain throughout the rearing period and the final body weight of 16-week-old turkeys were significantly improved when the dietary Met level was increased from 30% to 45% of Lys content and the Arg level was increased from 90% to 100% of Lys content. Moreover, the higher inclusion levels of these amino acids were effective in improving the immune and antioxidant status of the birds. As reported by Rivera-Torres et al. [32], the Arg content of feather protein in modern commercial farming systems of turkeys is equally as high as the Cys content (approximately 7.0%). Despite the aforementioned data, the relationship between dietary Arg and feathering remains insufficiently investigated. Kessler and Thomas [33] used growth and feed efficiency as well as molted body feathers (feather loss) to estimate the Arg requirements of 4- to 7-week-old broilers. Wylie et al. [11] reported that Arg added to a protein-deficient diet for young turkeys (2 to 6 weeks of age) was preferentially directed to feather growth rather than muscle growth which contributed to a significant increase in feather weight.

We hypothesized that different ratios of dietary Met and Arg could affect feather growth in young turkeys. The present study aimed to further expand our knowledge and investigate the effects of different dietary Arg levels, at low and high Met levels, and at low dietary Lys content consistent with NRC [34] recommendations, on plumage development in 4- and 16-week-old female turkeys.

## 2. Materials and Methods

### 2.1. Animals and Housing Conditions

This study is part of a multi-aspect experiment conducted by two research teams on the experimental poultry farm at the University of Warmia and Mazury in Olsztyn. The details regarding the applied nutrition program, management, and housing conditions as well as the growth performance, carcass traits, antioxidant status, and immune response of turkeys have been described previously by Jankowski et al. [27,28,29] and Ognik et al. [30,31].

Briefly, a total of 864 one-day-old Hybrid Converter female turkey poults purchased from a local hatchery were randomly assigned to six dietary treatment groups, with eight replicate pens (2.0 m × 2.0 m, 4 m^2^ each) per group and 18 birds per pen. Floor pens were bedded with wood shavings. The turkeys were raised to 16 weeks of age, under identical environmental conditions that were controlled automatically and adapted to the birds’ age. Throughout the experiment, animals had unrestricted access to feed and water.

### 2.2. Experimental Design and Diets

The birds were fed six diets in a 2 × 3 factorial arrangement, with two dietary Met levels (0.30% and 0.45% relative to Lys content) and three Arg levels (90%, 100%, and 110% relative to Lys content). The dietary treatment groups were denoted as follows: Met_30_Arg_90_, Met_45_Arg_90_, Met_30_Arg_100_, Met_45_Arg_100_, Met_30_Arg_110_, and Met_45_Arg_110_. Isocaloric experimental diets contained 1.6%, 1.5%, 1.3%, and 1.0% Lys, as recommended by the NRC [34], and were administered to turkeys at 1–4, 5–8, 9–12, and 13–16 weeks of age, respectively. According to the experimental procedure, basal diets without supplemental Lys, Met, or Arg were prepared for each of the four feeding periods of four weeks each (Table S1). The amino acid content of basal diets was determined analytically, and then they were mixed with the appropriate amounts of the pure amino acids L-lysine, L-arginine, and DL-methionine. The actual levels of supplemental Lys, Arg, and Met in experimental diets were obtained by adding L-Lys HCL, L-Arg HCl, and DL-Met to the basal diet. The Cys content of the diets was constant, and the source of Cys was the basal diet. The analyzed content of this amino acid in the basal diets was approximately 0.46, 0.41, 0.38, and 0.33% in successive months of the experiment. The total sulfur amino acid (TSAA, Met + Cys) content of complete diets with low and high Met levels was approximately 60% and 75% relative to Lys, respectively. The results of analysis of all tested diets, as well as the amount of amino acids added to the basal diets can be found in Supplementary Materials (Tables S2 and S3).

### 2.3. Feather Collection and Analysis

Forty-eight 4-week-old female turkeys (eight turkeys per group) and the same number of birds euthanized at 16 weeks of age were available as part of the aforementioned study by Jankowski et al. (2020a). In order to evaluate feather growth, samples of feathers were collected from the outer side of one thigh and from an area of 4 cm^2^ in the interscapular region, 2 cm^2^ on both sides of the spine. These two regions were analyzed because they are characterized by a different rate of feathering, which is fast in the interscapular region and slow in the thigh region [24,35]. A total of 192 feather-pooled samples (32 samples from each treatment) were collected. In each sample, the longest and the shortest mature feathers were measured to determine their average length. The feathers were then dried for 72 h at room temperature, and classified into five growth stages: I—pinfeathers covered in sheaths, II—beginning of vane development, III—feathers unsheathed by ½ of rachis length, IV—feathers unsheathed by ¾ of rachis length, and V—mature feathers, according to the previously presented methods [24,35]. The pattern of thigh and interscapular feathers representing different growth stages in a 4- and 16-week-old female turkeys is presented in Section 3.1. The total number of feathers collected from the dorsal and thigh regions was determined, and the percentage of feathers in each growth stage was calculated.

### 2.4. Statistical Analysis

The data on feather characteristics in different growth stages were analyzed in a 2 × 3 factorial design by two-way ANOVA with the general linear model (GLM) procedure of STATISTICA software version 13.1 (TIBCO Inc., Palo Alto, USA) [36]. An individual bird (*n* = 8) was considered as the experimental unit in analyses of feather characteristics. For statistical comparisons, percentage values were converted using the asin(sqrt(×/100) function. The main effects of dietary Met level, Arg level, and their interaction for each parameter were determined. The proportions of thigh and interscapular feathers in different growth stages, analyzed in combination and separately, were included in the model. When the model was significant, Tukey’s test was used to separate treatment means. All data are presented as means with the pooled standard error of the mean (SEM), and differences were considered statistically significant at *p* < 0.05.

### 2.5. Ethical Statement

The experiment was approved by the Ethics Committee of the University of Warmia and Mazury (ID: 82/2017). The animals were cared for under guidelines comparable to those laid down by EU Directive 2010/63/EU.

## 3. Results

### 3.1. General Characteristics of Thigh and Dorsal Feathers

**Figure 1:**
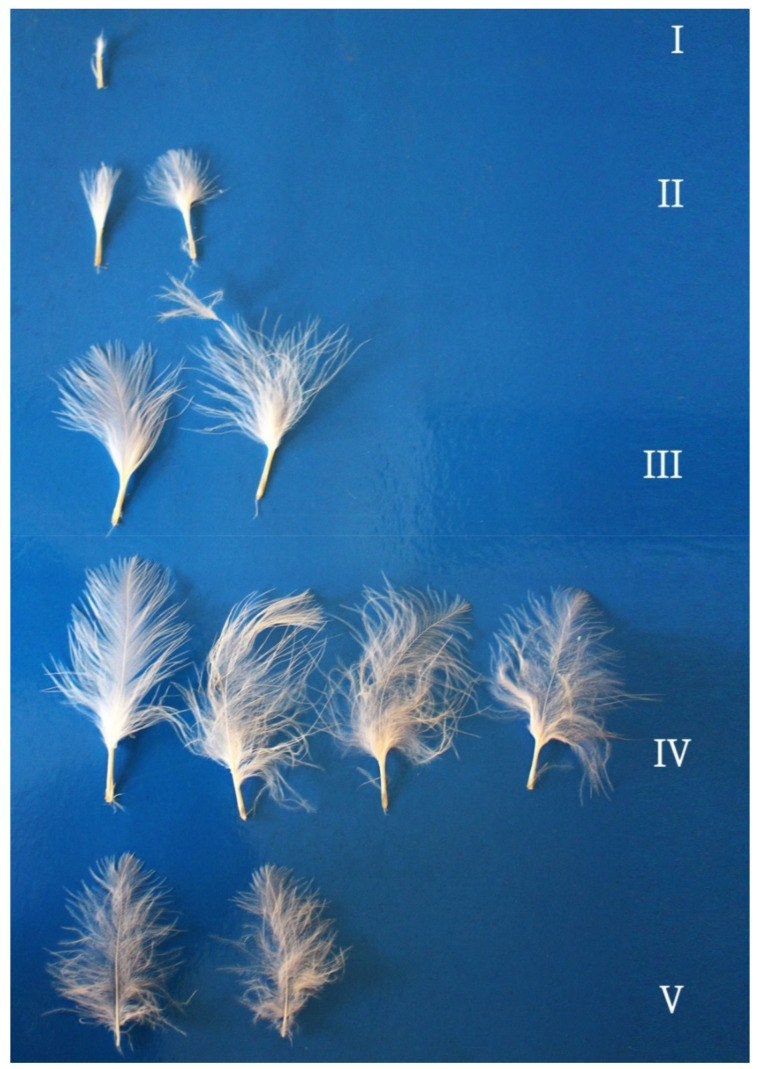
Thigh feathers of a 4-week-old turkey. Feather growth stages: I—pinfeather covered in sheath; II– barbs protruding from the sheath; III—plumulaceous (loose) vane (thigh), pennaceous (coherent) vane (interscapular region), feathers unsheathed by ½ of rachis length; IV—plumulaceous (loose) vane (thigh), pennaceous (coherent) vane (interscapular region), feathers unsheathed by ¾ of rachis length; V—mature feathers, feather length: shortest—2.5 cm, longest—3.0 cm, absence of afterfeathers and empty spaces between barbs.

**Figure 2:**
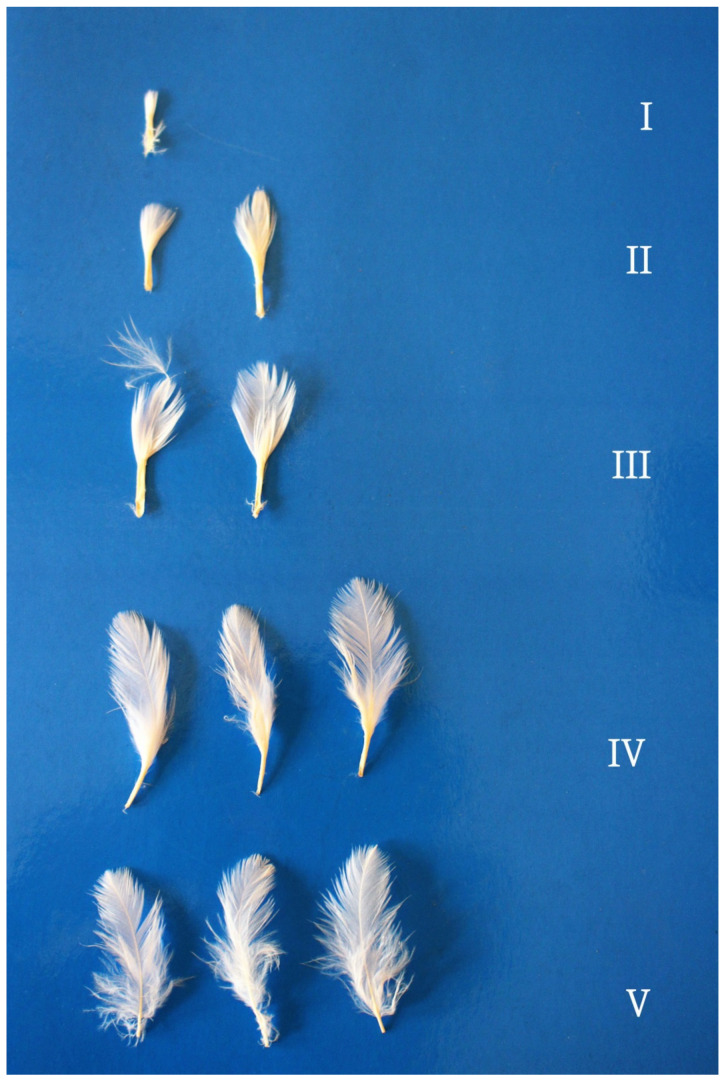
Interscapular feathers in a 4-week-old turkey. Feather growth stages: I—pinfeather covered in sheath; II– barbs protruding from the sheath; III—plumulaceous (loose) vane (thigh), pennaceous (coherent) vane (interscapular region), feathers unsheathed by ½ of rachis length; IV—plumulaceous (loose) vane (thigh), pennaceous (coherent) vane (interscapular region), feathers unsheathed by ¾ of rachis length; V—mature feathers, feather length: shortest—2.5 cm, longest—3.0 cm, absence of afterfeathers and empty spaces between barbs.

A plumage pattern in 4-week-old turkeys is shown in Figure 1 and Figure 2. Some thigh and interscapular feathers had down feathers that had not been shed. The feathers of 4-week-old birds consisted of a calamus, rachis, and vane. Thigh feathers in all growth stages had soft vanes along the entire length of the rachis (Figure 1). Interscapular feathers had coherent (pennaceous coherent) vanes, and loose (plumulaceous) vanes were observed only in stage V feathers (Figure 2). The length of mature feathers in 4-week-old turkeys was 2.5 to 3.0 cm in both regions.

**Figure 3:**
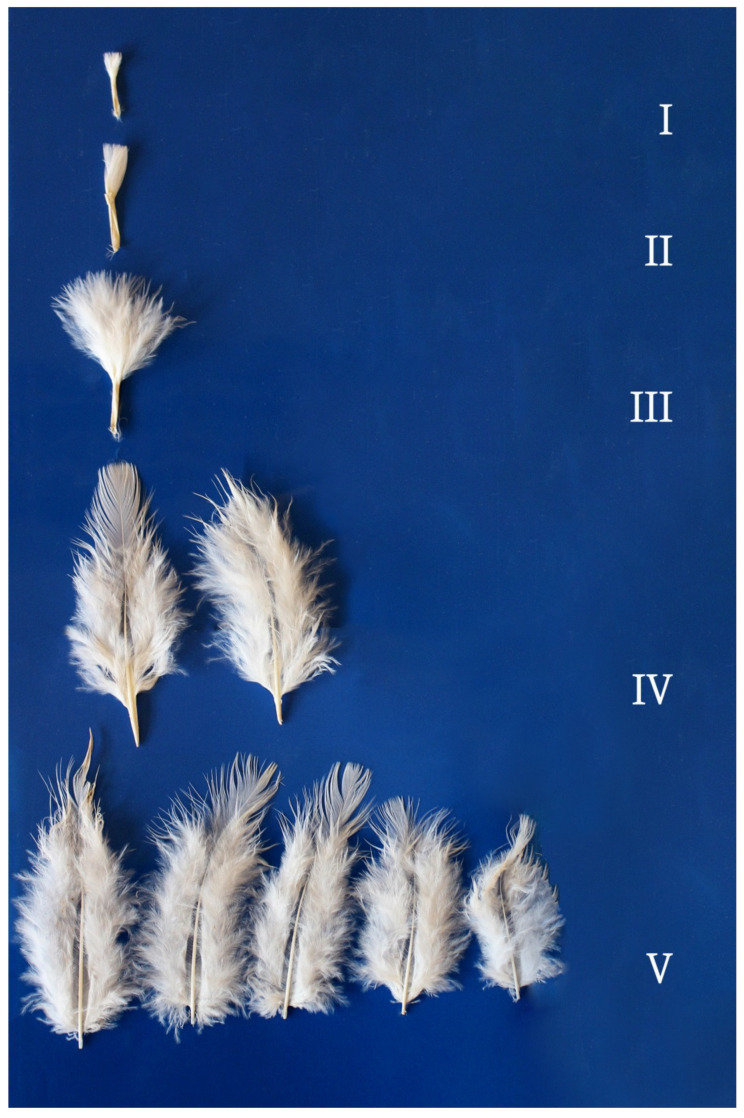
Thigh feathers in a 16-week-old turkey. Feather growth stages: I—pinfeather covered in sheath; II—barbs protruding from the sheath; III—feather unsheathed by ½ of rachis length; IV—plumulaceous (loose) vane and pennaceous (coherent) vane, feathers unsheathed by ¾ of rachis length; V—mature feathers, feather length: shortest—3.5 cm, longest—5.0 cm, afterfeathers and empty spaces between barbs.

**Figure 4:**
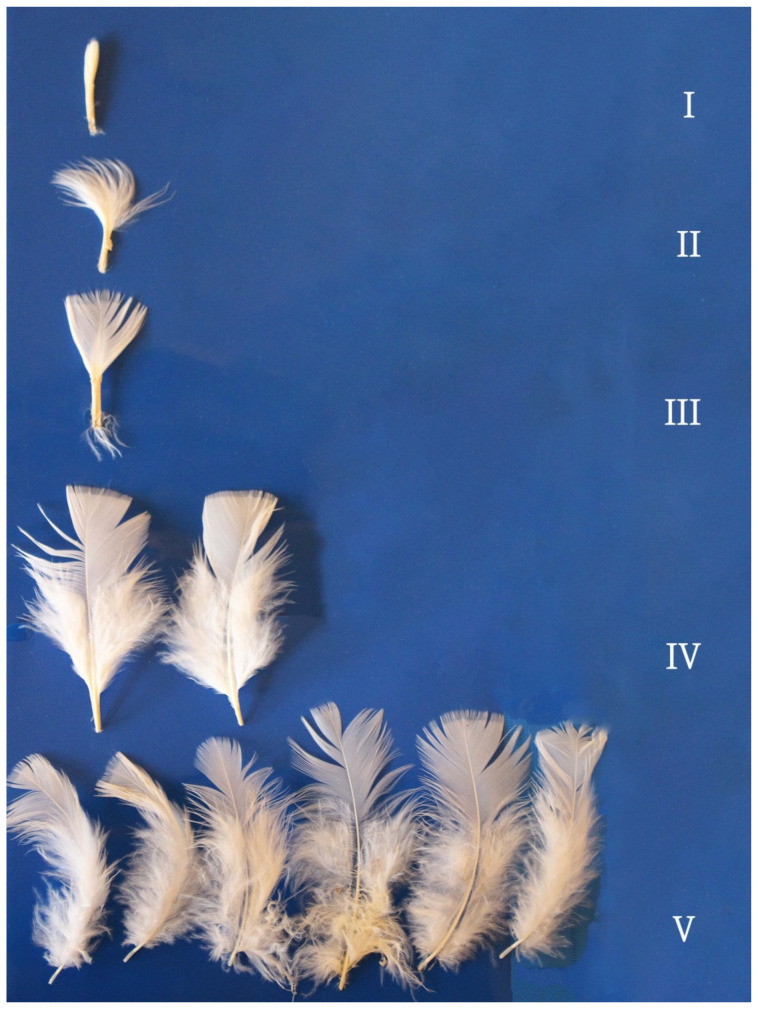
Interscapular feathers in a 16-week-old turkey. Feather growth stages: I—pinfeather covered in sheath; II—barbs protruding from the sheath; III—feather unsheathed by ½ of rachis length; IV—plumulaceous (loose) vane and pennaceous (coherent) vane, feathers unsheathed by ¾ of rachis length; V—mature feathers, feather length: shortest—3.5 cm, longest—5.0 cm, afterfeathers and empty spaces between barbs.

Sixteen-week-old turkeys had feathers in all growth stages (Figure 3 and Figure 4). Additional morphological structures such as afterfeathers and empty spaces between barbs without barbules on both sides of the rachis were noted in stage IV and stage V feathers, but they were absent in earlier growth stages. Afterfeathers growing out of the calamus were clearly visible in interscapular feathers, and they were very short in thigh feathers. Each afterfeather had a rachis and a loose vane. Coherent vanes were found in stage IV and stage V feathers on the thigh. The length of mature feathers in 16-week-old turkeys was 3.5 to 5.0 cm in both regions.

The average number of feathers in the interscapular and thigh regions is presented in Table 1. In 4-week-old female turkeys, the interscapular region was characterized by a higher total number of feathers than the thigh region (63 vs. 41), and a higher number of mature feathers (18 vs. 7) as well as feathers in successive growth stages. The number of feathers in the dorsal region did not increase between weeks 4 and 16 (63.2–63.5), whereas the number of feathers on the thigh nearly doubled (40.6–75.1). Sixteen-week-old turkeys had the highest number of stage V feathers (38.77–48.37), and the lowest number of stage I feathers (0.72–0.81), in both regions.

### 3.2. The Effect of Dietary Met and Arg Levels

Until 4 weeks of age, graded dietary inclusion levels of Arg and Met had no influence on the percentage of thigh and interscapular feathers in different growth stages, analyzed separately or in combination (Table 2 and Table S4). Moreover, no significant interactions between Met and Arg concentrations were observed for any of the growth stages. The proportion of stage I thigh and interscapular feathers (combined analysis) ranged from 7.63% to 10.26% at different Arg levels, and from 8.20% to 9.37% at both Met concentrations. The percentages of feathers in later growth stages were higher; mature feathers accounted for 22.92% to 25.83% of all feathers at different Arg levels, and for 22.55–26.18% at both Met concentrations. A lower percentage of stage IV feathers and a higher percentage of stage V feathers indicated an earlier molting in the interscapular compared with the thigh region (Table 2).

Significant differences in plumage development, resulting from different dietary inclusion levels of Met, were found in 16-week-old turkeys (Table 3 and Table S5). An increase in the Met inclusion rate to 45% of Lys content accelerated plumage development, which was confirmed in a combined analysis by a lower percentage of stage IV feathers and a higher proportion of mature feathers in stage V (both *p* = 0.001; Table S5). As shown in Table 3, these differences were significant only in the thigh region. The higher inclusion rate of Met (45% to Lys) strongly accelerated feather growth on the thigh (*p* = 0.001), whereas the increase in the percentage of stage V (mature) feathers in the interscapular region was less pronounced (*p* = 0.074), compared with the thigh region. Turkeys fed diets with increased Met content were characterized by a considerably lower percentage of stage II (*p* = 0.035), stage III (*p* = 0.019), and stage IV (*p* = 0.003) feathers, and a higher percentage of stage V (mature) feathers (*p* = 0.001) on the thigh.

Different dietary Arg concentrations had no effect on plumage development in 16-week-old turkeys, when thigh and interscapular feathers were analyzed separately and in combination (Table 3 and Table S5). The percentage of stage II feathers in the interscapular region was lowest in birds receiving diets with the highest Arg concentration (110% relative to Lys content, *p* < 0.046). However, it had no direct influence on the percentage of stage V (mature) feathers and higher Arg levels did not significantly accelerate feather maturation in this body region.

## 4. Discussion

### 4.1. General Characteristics of Turkey Feathers

Plumage development in the analyzed turkeys was typical of gallinaceous birds [37]. The rate of feather growth was slower on the thigh and faster in the interscapular region. A higher number of feathers in the interscapular region, compared with the thigh, was also noted in previous studies regarding Galliformes [24,35]. The proportion of mature feathers was higher in the interscapular region where molting most likely began at around 4 weeks of age. Leeson and Walsh [12] reported that the second molt in broiler chickens usually begins at around 4–5 weeks of age, but they did not specify in which region of the body.

Feathers from the analyzed body regions differed in structure. Thigh feathers were soft and had very loose vanes; coherent vanes were noted only in some feathers in growth stages IV and V. Mature feathers in the dorsal region had coherent and loose vanes in turkeys aged 4 and 16 weeks. Afterfeathers and empty spaces between barbs were observed in thigh and interscapular feathers in 16-week-old birds. Afterfeathers were short and differed from the feathers of sexually mature turkeys. In Green-legged Partridge chickens, afterfeathers and empty spaces between barbs in mature feathers in the dorsal and thoracic regions had similar lengths at 12 weeks of age [38]. The thigh and dorsal feathers of guinea-fowl had coherent and loose vanes, afterfeathers, and empty spaces between barbs of similar lengths, also at 12 weeks of age. The additional morphological structures develop faster in chickens and guinea-fowl than in turkeys [35,38]. Somatic development takes longer in turkeys than in chickens and guinea-fowl, which leads to differences in feather structure [37]. It is difficult to compare plumage development in turkeys and other bird species due to the use of different research methods [4,11,14,19,23,39].

### 4.2. The Effect of Dietary Met and Arg Levels

Feather development consisting of five growth stages was the main criterion for determining the effects of dietary Met and Arg levels in this study. The number of feathers in the thigh and interscapular regions, presented in this study, constituted input data that were used for analysis of the proportions of feathers in different growth stages (Table 2 and Table 3,  Tables S4 and S5). Differences in the number of feathers in the examined body regions under the influence of experimental diets were not expected. It is known that feather primordia are formed in the embryonic stage [40].

Experiments performed on different poultry species have demonstrated that birds need both Met and Cys to synthesize protein, therefore TSAA levels are an important limiting factor [20,21,22,41,42]. The results of the current study indicate that an increased supply of sulfur-containing amino acids via supplemental Met can contribute to promoting feather growth in young turkeys, which had not been confirmed in a previous study [24]. Zeng et al. [19] found both improved growth performance and a significant increase in feather cover (infrared images) and feather length in 35-day-old ducks in 0.45% and 0.56% Met groups compared with those receiving 0.30% and 0.39% of Met. In turn, Zhao et al. [23] demonstrated that dietary Met supplementation increased feather weight in ducks. Methionine injected in ovo increased the density and diameter of chicken-embryo feather follicles [43]. Methionine has been found to promote follicle and feather growth in developing chick embryos by activating the Wnt/β-catenin signaling pathway [44,45].

In the present study, the absence of plumage response to increased dietary Met content, noted in 4-week-old turkeys, could suggest that their general maintenance and muscle growth requirements were greater than their feather development needs. The higher inclusion levels of Met and Arg were effective in improving the immune and antioxidant status of the turkeys [25,26,27,30]. Mounting an immune response is energetically costly for birds, and it may divert energy from feather growth. These results are partially consistent with the findings of Kubińska et al. [24] who reported that supplemental Met improved body weight gain in 8-week-old turkeys, but it had no effect on plumage development at 4 and 8 weeks of age.

In the current study, the positive response to increased TSAA levels, reflected in feather growth, was observed only in 16-week-old turkeys. Jankowski et al. [27] found that throughout the rearing period, the higher dietary Met level (45% vs. 30% of Lys content) increased body weight gains and the final body weight of 16-week-old turkeys, whereas Arg levels (100% and 110 vs. 90% of Lys content) had no influence on their growth performance. It is also noteworthy that the higher Met level (45% vs. 30% of Lys content) positively affected energy and protein utilization, and improved Arg and Lys retention efficiency in 4-week-old turkeys [46].

Similarly to Met, the dietary Arg:Lys ratio also plays an important role in the body functioning [27,28,29,30,31,39]. Jankowski et al. [27] demonstrated that at the low level of dietary Met (30% relative to Lys), a decrease in Arg relative to Lys from 100% to 90% caused a noticeable growth depression in turkeys. Moreover, the lowest Arg level (90% of Lys content) undesirably increased the concentration of the proinflammatory cytokine IL-6, decreased globulin concentration in the blood plasma, and decreased the dressing yield of turkeys [27]. In turn, Ognik et al. [30] reported that high-Met diets containing 90% Arg relative to Lys contributed to minimizing oxidative stress and modulating metabolic parameters and intestinal-barrier parameters in turkeys with necrotizing enterocolitis. However, no information was found in the available literature regarding the effects of dietary Arg:Lys ratios on feather growth in modern commercial poultry farming systems. In the present study, the dietary supply of Arg did not promote the growth of thigh feathers or interscapular feathers in turkeys aged 4 and 16 weeks. The absence of response observed in turkeys fed diets with varying Arg levels suggests that 90% Arg relative to Lys content was adequate for feathering. According to Rivera-Torres et al. [32], the Arg content of feather protein is high (approx. 7.0%) but relatively stable during the growth period of turkeys. It is therefore likely that the Arg requirement for feather growth does not change in growing turkeys. As a result, the increase in Arg supply did not affect feather growth in this study.

## 5. Conclusions

The results of this study indicate that the administration of diets with an increased Met level corresponding to 45% of Lys content contributes to optimizing plumage development in young turkeys. The increased dietary Met content (from 30% to 45% relative to Lys content) at low Lys content, consistent with NRC (1994) recommendations, accelerated the development of thigh feathers, which was confirmed by an increase in the proportion of mature feathers in this region in 16-week-old turkeys. Data on feather growth can contribute to determining the TSAA requirements of growing turkeys. Different concentrations of Arg (90%, 100% and 110% of Lys content) had no influence on plumage development in turkeys at 4 and 16 weeks of age.

## Figures and Tables

**Table 1:** Average number of feathers in the analyzed body regions of turkeys.

Feather Growth Stage	Thigh Region		Interscapular Region	
	Week 4	Week 16	Week 4	Week 16
I. Pinfeathers covered in sheaths	3.22	0.72	5.66	0.81
II. Beginning of vane development	6.60	2.20	10.50	2.50
III. Feathers unsheathed by ½ of rachis length	8.27	5.75	13.47	4.58
IV. Feathers unsheathed by ¾ of rachis length	15.60	18.10	16.33	16.52
V. Mature feathers	6.93	48.37	17.58	38.77
Total number of feathers	40.62	75.14	63.54	63.18

**Table 2:** Proportions of thigh and interscapular feathers (separately) in different growth stages in 4-week-old turkeys.

Item	Average Number of Feathers	Feather Growth Stage in Thigh Region ^1^					Average Number of Feathers	Feather Growth Stage in Interscapular Region ^1^				
		I	II	III	IV	V		I	II	III	IV	V
Treatment (*n* = 8) ^2^												
Arg90Met30	34.50	7.12	15.22	16.45	43.32	17.89	62.63	9.42	14.52	18.33	25.67	32.06
Arg90Met45	39.25	7.14	19.43	22.34	35.01	16.09	64.88	9.43	18.57	23.85	26.73	21.42
Arg100Met30	43.25	11.83	13.36	24.25	31.72	18.84	53.38	10.16	13.09	25.50	24.12	27.13
Arg100Met45	44.38	7.20	16.82	18.38	42.58	15.02	59.88	9.31	14.89	20.81	28.12	26.87
Arg110Met30	41.88	9.45	15.89	19.58	37.71	17.36	70.25	7.44	19.47	16.85	25.32	30.92
Arg110Met45	40.63	7.09	15.30	20.10	38.09	19.41	70.38	7.35	21.14	20.97	23.58	26.95
SEM	1.892	1.048	1.535	1.535	1.736	1.107	3.212	0.777	1.227	1.224	1.098	1.559
Arginine, %												
90	36.88	7.13	17.33	19.39	39.16	16.99	63.75	9.43	16.54	21.09	26.20	26.74
100	43.81	9.51	15.09	21.32	37.15	16.93	56.63	9.73	13.99	23.16	26.12	27.00
110	41.25	8.27	15.59	19.84	37.90	18.39	70.31	7.39	20.31	18.91	24.45	28.94
Methionine, %												
30	39.88	9.47	14.82	20.09	37.58	18.03	62.08	9.00	15.69	20.23	25.04	30.04
45	41.42	7.14	17.18	20.27	38.56	16.84	63.04	8.70	18.20	21.88	26.14	25.08
*p*-value												
Arg	0.345	0.460	0.821	0.878	0.768	0.871	0.244	0.398	0.167	0.390	0.580	0.781
Met	0.693	0.542	0.229	0.930	0.610	0.490	0.654	0.821	0.226	0.506	0.936	0.153
Arg × Met interaction	0.817	0.677	0.546	0.367	0.087	0.491	0.921	0.972	0.786	0.167	0.505	0.390

^1^ Growth stage of feathers: I—pinfeathers covered in sheaths, II—beginning of vane development, III—feathers unsheathed by ½ of rachis length, IV—feathers unsheathed by ¾ of rachis length, and V—mature feathers. ^2^ Treatment: Arg90Met30 received 90% Arg level and 30% Met level relative to the content of dietary Lys; Arg90Met45 received 90% Arg level and 45% Met level relative to the content of dietary Lys; Arg100Met30 received 100% Arg level and 30%Met level relative to the content of dietary Lys; Arg100Met45 received 100% Arg level and 45% Met level relative to the content of dietary Lys; Arg110Met30 received 110% Arg level and 30% Met level relative to the content of dietary Lys; Arg110Met45 received 110% Arg level and 45% Met level relative to the content of dietary Lys.

**Table 3:** Proportions of thigh and interscapular feathers (separately) in different growth stages in 16-week-old turkeys.

Item	Average Number of Feathers	Feather Growth Stage in Thigh Region ^1^					Average Number of Feathers	Feather Growth Stage in Interscapular Region ^1^				
		I	II	III	IV	V		I	II	III	IV	V
Treatment (*n* = 8) ^2^												
Arg90Met30	76.12	0.53	3.63	8.54	32.97 ^a^	54.34	55.50	1.25	4.96	6.94	27.77	59.08
Arg90Met45	78.38	1.34	3.16	7.41	16.22 ^b^	71.86	59.50	1.37	5.21	9.17	22.05	62.20
Arg100Met30	77.50	1.32	3.03	10.70	24.60 ^ab^	60.35	63.50	2.71	3.94	8.35	26.52	58.49
Arg100Met45	72.13	1.23	2.61	5.04	21.51 ^ab^	69.60	65.50	1.14	3.78	6.36	24.32	64.41
Arg110Met30	72.38	1.42	4.28	7.86	23.94 ^ab^	62.49	70.13	0.38	3.99	5.77	30.61	59.25
Arg100Met45	74.37	0.16	0.39	3.64	21.40 ^ab^	74.42	65.00	1.08	1.31	5.37	27.65	64.59
SEM	1.640	0.246	0.417	0.815	1.328	1.771	1.985	0.287	0.449	0.630	1.186	1.312
Arginine, %												
90	77.25	0.94	3.40	7.98	24.59	63.10	57.50	1.31	5.08 ^a^	5.08	24.91	60.64
100	74.81	1.28	2.82	7.87	23.06	64.98	64.50	1.92	3.86 ^ab^	3.86	25.42	61.45
110	73.37	0.79	2.34	5.75	22.67	68.45	67.56	0.73	2.65 ^b^	2.65	29.13	61.92
Methionine, %												
30	75.33	1.09	3.65 ^a^	9.03 ^a^	27.17 ^a^	58.06 ^b^	63.04	1.45	4.29	7.02	28.30	58.94
45	74.96	0.91	2.05 ^b^	5.37 ^b^	19.71 ^b^	71.96 ^a^	63.33	1.20	3.43	6.97	24.67	63.73
*p*-value												
Arg	0.644	0.441	0.323	0.347	0.875	0.345	0.115	0.223	0.046	0.348	0.289	0.911
Met	0.912	0.661	0.035	0.019	0.003	0.001	0.941	0.739	0.303	0.852	0.111	0.074
Arg × Met interaction	0.584	0.315	0.133	0.225	0.030	0.561	0.615	0.333	0.142	0.373	0.800	0.900

^1^ Growth stage of feathers: I—pinfeathers covered in sheaths, II—beginning of vane development, III—feathers unsheathed by ½ of rachis length, IV—feathers unsheathed by ¾ of rachis length, and V—mature feathers. ^2^ Treatment: Arg90Met30 received 90% Arg level and 30% Met level relative to the content of dietary Lys; Arg90Met45 received 90% Arg level and 45% Met level relative to the content of dietary Lys; Arg100Met30 received 100% Arg level and 30%Met level relative to the content of dietary Lys; Arg100Met45 received 100% Arg level and 45% Met level relative to the content of dietary Lys; Arg110Met30 received 110% Arg level and 30% Met level relative to the content of dietary Lys; Arg110Met45 received 110% Arg level and 45% Met level relative to the content of dietary Lys. ^a,b^ Means within the same column with different superscripts differ significantly (*p* < 0.05).

## Data Availability

All data generated or analyzed during the study are included in this published article or supplementary material. The datasets used and/or analyzed in the current study are available from the corresponding author on reasonable request.

## Supplementary Materials

The following are available online at https://www.mdpi.com/article/10.3390/ani12020172/s1, Table S1: Ingredient composition and nutrient content of basal diets (g/100 g, as-fed basis). Table S2: Amino acids added to basal diets (g/100 g). Table S3: Analyzed total amino acid contents of the experimental diets (%). Table S4: Proportions of thigh and interscapular feathers (combined) in different growth stages in 4-week-old turkeys. Table S5: Proportions of thigh and interscapular feathers (combined) in different growth stages in 16-week-old turkeys.
