# Supplementary material for: Plumage Response of Young Turkeys to Diets with Increased Methionine to Lysine Ratios at Three Dietary Arginine Levels
Source: Animals (Basel). 2022 Jan 12;12(2):172. doi: 10.3390/ani12020172 (PMC8772563; doi:10.3390/ani12020172)
Supplement: Supplementary file 1 [file animals-12-00172-s001.zip › animals-1506225-supplementary.pdf]

Table S1. Ingredient composition and nutrient content of basal diets (g/100 g, as-fed basis) [1].

Item	Feeding Period, Weeks			
	1 – 4	5 – 8	9 – 12	13 – 16
Ingredients				
Wheat	46.37	48.67	53.74	65.63
Maize	10.00	10.00	10.00	10.00
Soybean meal	25.05	23.27	18.73	7.91
Rapeseed meal	3.00	5.00	7.18	7.00
Potato protein	5.52	3.01	-	-
Soybean oil	0.20	2.32	3.53	3.22
Maize gluten meal	5.50	3.50	3.50	3.50
Sodium bicarbonate	0.20	0.20	0.20	0.20
Sodium chloride	0.15	0.16	0.16	0.14
Limestone	2.20	1.86	1.64	1.38
Monocalcium phosphate	1.46	1.29	0.90	0.50
L-Threonine	-	0.07	0.07	0.17
Choline chloride	0.10	0.10	0.10	0.10
Vitamin-mineral premix [2]	0.25	0.25	0.25	0.25
Titanium oxide	-	0.30	-	-
Calculated nutrient content				
Metabolizable energy, kcal/kg	2 825	2 900	3 000	3 100
Crude protein	26.50	23.50	20.50	17.00
Lysine total	1.28	1.12	0.89	0.64
Arginine total	1.44	1.35	1.17	0.89
Methionine total	0.45	0.39	0.34	0.29
Methionine + Cysteine total	0.92	0.82	0.74	0.65
Threonine total	1.02	0.95	0.80	0.75
Calcium	1.25	1.10	0.95	0.75
Available phosphorus	0.65	0.55	0.47	0.38

[1] Source: This table was published in Poultry Science, 99 Jan Jankowski, Dariusz Mikulski, Marzena Mikulska, Katarzyna Ognik, Zuzanna Całyniuk, Emilia Mróz, Zenon Zduńczyk, The effect of different dietary ratios of arginine, methionine, and lysine on the performance, carcass traits, and immune status of turkeys, 1028–1031, Copyright © Poultry Science Association. Published by Elsevier Inc. All rights reserved (2020). [2] Provided per kg of diet (feeding periods: weeks 1 – 4, 5 – 8, 9 – 12 and 13 – 16): mg: all-trans retinol 3.78, 3.38, 2.88 and 2.52; cholecalciferol 0.13, 0.12, 0.10 and 0.09; α-tocopheryl acetate 100, 90, 80 and 70; vit. K_3 5.8, 5.6, 4.8 and 4.2; thiamine 5.4, 4.7, 4.0 and 3.5; riboflavin 8.4, 7.5, 6.4 and 5.6; pyridoxine 6.4, 5.6, 4.8 and 4.2; cobalamin 0.032, 0.028, 0.024 and 0.021′ biotin 0.32, 0.28, 0.24 and 0.21; pantothenic acid 28, 24, 20 and 18; nicotinic acid 84, 75, 64 and 56; folic acid 3.2, 2.8, 2.4 and 2.1; Fe 64, 60, 56, 48 and 42; Mn 120, 112, 96 and 84; Zn 110, 103, 88 and 77; Cu 23, 19, 16 and 14; I 3.2, 2.8, 2.4 and 2.1; Se 0.30, 0.28, 0.24 and 0.21, respectively.

Table S2. Amino acids added to basal diets (g/100g) [1].

Feeding Period, Weeks	Amino Acid	Treatment [2]					
		Arg90Met30	Arg90Met45	Arg100Met30	Arg100Met45	Arg110Met30	Arg110Met45
1—4	L-Lysine HCl [3]	0.40	0.40	0.40	0.40	0.40	0.40
	L-Arginine HCL [4]	0	0	0.13	0.13	0.28	0.28
	DL-Methionine [5]	0.03	0.27	0.03	0.27	0.03	0.27
5—8	L-Lysine HCl	0.40	0.40	0.40	0.40	0.40	0.40
	L-Arginine HCL	0	0	0.13	0.13	0.28	0.28
	DL-Methionine	0.06	0.29	0.06	0.29	0.06	0.29
9—12	L-Lysine HCl	0.44	0.44	0.44	0.44	0.44	0.44
	L-Arginine HCL	0	0	0.11	0.11	0.24	0.24
	DL-Methionine	0.05	0.25	0.05	0.25	0.05	0.25

	L-Lysine HCl	0.48	0.48	0.48	0.48	0.48	0.48
13—16	L-Arginine HCL	0	0	0.09	0.09	0.19	0.19
	DL-Methionine	0	0.12	0	0.12	0	0.12

[1] Source: This table was published in Poultry Science, 99 Jan Jankowski, Dariusz Mikulski, Marzena Mikulska, Katarzyna Ognik, Zuzanna Całyniuk, Emilia Mróz, Zenon Zduńczyk, The effect of different dietary ratios of arginine, methionine, and lysine on the performance, carcass traits, and immune status of turkeys, 1028–1031, Copyright © Poultry Science Association. Published by Elsevier Inc. All rights reserved (2020). [2] Actual levels of supplementary Lys, Arg and Met in experimental diets were obtained by adding supplementary L-Lys HCL, L-Arg HCl and DL-Met on top to the basal diet. [3] L-Lysine HCL (Ajinomoto Eurolysine S.A.S, Amiens, France, 780 g lysine /kg) was added to the basal diet to provide 1.60, 1.50, 1.30 and 1.00 g of Lys per 100 g of feed in four successive feeding periods, according to the nutrient requirements of turkeys (NRC, 1994). [4] L-Arginine HCL (Ajinomoto Eurolysine S.A.S, Amiens, France, 990 g arginine /kg) was added to the basal diet to provide 90%, 100% and 110% of Arg relative to the content of dietary Lys. [5] DL-Methionine (MetAMINO®, Evonik Degussa Gmbh, Essen, Germany, 990 g methionine/kg) was added to provide 30% and 45% of Met relative to the content of dietary Lys.

Table S3. Analyzed total amino acid contents of the experimental diets (%).

Feeding Period, Weeks	Amino Acid	Treatment [1]					
		Arg90Met30	Arg90Met45	Arg100Met30	Arg100Met45	Arg110Met30	Arg110Met45
1—4	Lysine	1.63	1.56	1.58	1.66	1.55	1.64
	Arginine	1.46	1.43	1.52	1.56	1.69	1.67
	Met + Cys (TSAA)	0.96	1.15	0.97	1.17	0.98	1.20
	TSAA : Lys ratio [2]	59.02	73.85	61.52	70.60	63.35	73.29
	Met : TSAA ratio [2]	51.97	59.90	52.47	60.58	52.95	61.56
5—8	Lysine	1.48	1.45	1.53	1.56	1.56	1.55
	Arginine	1.37	1.39	1.53	1.56	1.71	1.73
	Met + Cys (TSAA)	0.83	1.07	0.83	1.11	0.85	1.07
	TSAA : Lys ratio [2]	56.01	73.72	54.18	71.09	54.42	68.97
	Met : TSAA ratio [2]	50.66	61.74	50.66	63.12	51.82	61.74
9—12	Lysine	1.27	1.29	1.34	1.28	1.36	1.32
	Arginine	1.18	1.20	1.34	1.26	1.45	1.43
	Met + Cys (TSAA)	0.76	0.99	0.79	0.97	0.76	0.97
	TSAA : Lys ratio [2]	59.61	76.51	58.73	75.55	55.66	73.26
	Met : TSAA ratio [2]	50.20	61.80	52.10	61.01	50.20	61.01
13—16	Lysine	1.01	0.99	1.03	1.04	0.96	0.97
	Arginine	0.92	0.91	1.05	1.03	1.12	1.16
	Met + Cys (TSAA)	0.66	0.79	0.64	0.75	0.65	0.81
	TSAA : Lys ratio [2]	65.74	80.20	62.52	72.5	68.12	83.92
	Met : TSAA ratio [2]	49.70	57.93	48.14	55.70	48.93	58.97

TSAA = Total sulfur amino acid. [1] Treatment: Arg90Met30 received 90% Arg and 30% Met relative to the content of dietary Lys; Arg90Met45 received 90% Arg and 45% Met relative to the content of dietary Lys; Arg100Met30 received 100% Arg and 30% Met relative to the content of dietary Lys; Arg100Met45 received 100% Arg and 45% Met relative to the content of dietary Lys; Arg110Met30 received 110% Arg and 30% Met relative to the content of dietary Lys; Arg110Met45 received 110% Arg level and 45% Met level relative to the content of dietary Lys. [2] Calculated.

Table S4. Proportions of thigh and interscapular feathers (combined) in different growth stages in 4-week-old turkeys.

Item	Average Number of Feathers	Feather Growth Stage [1]				
		I	II	III	IV	V
Treatment (n = 8) [2]						
Arg90Met30	97.13	8.84	14.69	17.85	29.55	29.06
Arg90Met45	104.13	9.17	18.66	23.37	29.17	19.62
Arg100Met30	96.63	12.20	12.48	24.71	27.24	23.36
Arg100Met45	104.25	8.31	14.57	21.95	32.69	22.48

Arg110Met30	112.13	8.15	19.45	17.94	28.35	26.11
Arg110Met45	111.0	7.11	19.37	21.05	26.91	25.55
SEM	4.435	0.637	1.020	1.102	1.060	1.152
Arginine, %						
90	100.63	9.01	16.68	20.61	29.36	24.34
100	100.44	10.26	13.53	23.33	29.97	22.92
110	111.56	7.63	19.41	19.50	27.63	25.83
Methionine, %						
30	101.96	9.37	15.54	20.17	28.38	26.18
45	106.46	8.20	17.54	22.12	29.59	22.55
P value						
Arg	0.533	0.238	0.116	0.376	0.618	0.517
Met	0.627	0.257	0.234	0.407	0.641	0.103
Arg × Met interaction	0.910	0.389	0.548	0.285	0.372	0.226

[1] Growth stage of feathers: I – pinfeathers covered in sheaths, II – beginning of vane development, III – feathers unsheathed by ½ of rachis length, IV – feathers unsheathed by ¾ of rachis length, V – mature feathers. [2] Treatment: Arg90Met30 received 90% Arg level and 30% Met level relative to the content of dietary Lys; Arg90Met45 received 90% Arg level and 45% Met level relative to the content of dietary Lys; Arg100Met30 received 100% Arg level and 30%Met level relative to the content of dietary Lys; Arg100Met45 received 100% Arg level and 45% Met level relative to the content of dietary Lys; Arg110Met30 received 110% Arg level and 30% Met level relative to the content of dietary Lys; Arg110Met45 received 110% Arg level and 45% Met level relative to the content of dietary Lys.

Table S5. Proportions of thigh and interscapular feathers (combined) in different growth stages in 16-week-old turkeys.

**Item**	**Average Number of Feathers**	**Feather Growth Stage** [1]				
		**I**	**II**	**III**	**IV**	**V**
Treatment (n = 8) [2]						
Arg90Met30	131.63	0.67	3.93^{a}	9.02	31.73	54.64
Arg90Met45	137.88	1.34	3.84^{a}	8.39	18.71	67.72
Arg100Met30	141.00	1.90	3.70^{a}	9.55	25.72	59.13
Arg100Met45	137.63	1.13	3.07^{a}	5.80	22.42	67.58
Arg110Met30	142.50	1.06	4.57^{a}	7.39	27.03	59.96
Arg110Met45	139.38	0.59	0.93^{b}	4.97	23.63	69.88
SEM	2. 446	0.215	0.317	0.590	1.060	1.394
Arginine, %						
90	134.75	1.01	3.89	8.70	25.22	61.18
100	139.31	1.51	3.38	7.68	24.07	63.36
110	140.93	0.82	2.75	6.18	25.33	64.92
Methionine, %						
30	138.38	1.21	4.07^{a}	8.65	28.16^{a}	57.91^{b}
45	138.29	1.02	2.61^{b}	6.39	21.59^{b}	68.39^{a}
P value						
Arg	0.590	0.322	0.087	0.175	0.823	0.431
Met	0.986	0.767	0.010	0.068	0.001	0.001
Arg × Met interaction	0.679	0.450	0.008	0.369	0.065	0.748

[1] Growth stage of feathers: I – pinfeathers covered in sheaths, II – beginning of vane development, III – feathers unsheathed by ½ of rachis length, IV – feathers unsheathed by ¾ of rachis length, V – mature feathers. [2] Treatment: Arg90Met30 received 90% Arg level and 30% Met level relative to the content of dietary Lys; Arg90Met45 received 90% Arg level and 45% Met level relative to the content of dietary Lys; Arg100Met30 received 100% Arg level and 30%Met level relative to the content of dietary Lys; Arg100Met45 received 100% Arg level and 45% Met level relative to the content of dietary Lys; Arg110Met30 received 110% Arg level and 30% Met level relative to the content of dietary Lys; Arg110Met45 received 110% Arg level and 45% Met level relative to the content of dietary Lys. ^{a,b} Means within the same column with different superscripts differ significantly ($p < 0.05$).
